# A Review on Antifungal Efficiency of Plant Extracts Entrenched Polysaccharide-Based Nanohydrogels

**DOI:** 10.3390/nu13062055

**Published:** 2021-06-15

**Authors:** Navkiranjeet Kaur, Aarti Bains, Ravinder Kaushik, Sanju B. Dhull, Fogarasi Melinda, Prince Chawla

**Affiliations:** 1 Department of Food Technology and Nutrition, Lovely Professional University, Phagwara 144411, Punjab, India; navikooner1998@gmail.com; 2 Department of Biotechnology, Chandigarh Group of Colleges Landran, Mohali 140307, Punjab, India; aarti05888@gmail.com; 3 School of Health Sciences, University of Petroleum and Energy Studies, Dehradun 248007, Uttrakhand, India; ravinder_foodtech2007@rediffmail.com; 4 Department of Food Science and Technology, Chaudhary Devi Lal University, Sirsa 125055, Haryana, India; sanjudhull@gmail.com; 5 Department of Food Engineering, University of Agricultural Sciences and Veterinary Medicine of Cluj-Napoca, Calea Mănăstur 3–5, 400372 Cluj-Napoca, Romania

**Keywords:** fungal infections, nanohydrogel, skin, polysaccharide, essential oils

## Abstract

Human skin acts as a physical barrier; however, sometimes the skin gets infected by fungi, which becomes more severe if the infection occurs on the third layer of the skin. Azole derivative-based antifungal creams, liquids, or sprays are available to treat fungal infections; however, these formulations show various side effects on the application site. Over the past few years, herbal extracts and various essential oils have shown effective antifungal activity. Additionally, autoxidation and epimerization are significant problems with the direct use of herbal extracts. Hence, to overcome these obstacles, polysaccharide-based nanohydrogels embedded with natural plant extracts and oils have become the primary choice of pharmaceutical scientists. These gels protect plant-based bioactive compounds and are effective delivery agents because they release multiple bioactive compounds in the targeted area. Nanohydrogels can be applied to infected areas, and due to their contagious nature and penetration power, they get directly absorbed through the skin, quickly reaching the skin’s third layer and effectively reducing the fungal infection. In this review, we explain various skin fungal infections, possible treatments, and the effective utilization of plant extract and oil-embedded polysaccharide-based nanohydrogels.

## 1. Introduction

Skin acts as a protector of the internal organs by shielding against external agents, sunburn, and by regulating body temperature; however, sometimes pathogens invade the body and disturb the skin protective properties, leading to skin diseases or infections [1]. Bacteria, viruses, parasites, and fungi can cause skin diseases. Fungal infections are more severe because they occur on the third layer of the skin [2]. Fungi act on keratin tissue such as skin, nails, and hair [3]. In the skin, fungi lead to subcutaneous infections, and over the past years, the cases of fungal skin infections have been increasing rapidly, especially in immune-compromised individuals [4]. Several well-known severe skin infections (Table 1) such as Tinea corporis (ringworm), Tinea pedis, Tinea faciei, Tinea manuum, Tinea cruris (Jock-itch), and Tinea barbae are caused mainly by *Trichophyton* species [5,6].

**Figure 1:**
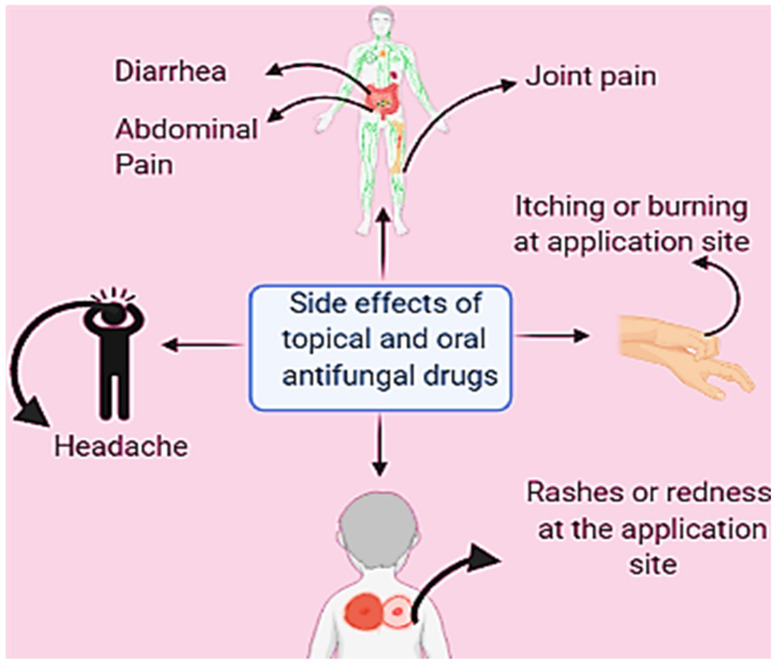
Side effects of synthetic antifungal drugs. These side effects are due to uncontrolled drug release and can lead to prolonged treatment due to low penetration; consequently, these drugs may not reach the target location and could lead to the incomplete clearance of infection.

Fungal infections are typically recognized by symptoms such as itchy red color patches, hair loss, and crusted patches [13]. Some common conditions leading to fungal infection are wearing tight-fitting clothes or sharing a locker room, clothes, or furniture with an infected person [14]. Antifungal drugs, primarily topical, oral, and intravenous, are used to treat various types of fungal infections; however, oral antifungal drugs are more toxic to the human body as compared to topical antifungal drugs. Additionally, commonly used antifungal drugs contain different types of broad categories of components such as azole, echinocandin, and polyenes [15]. Azoles inhibit the oxidative enzymes present in the fungal cell membrane, which prevents the cell wall of the fungus from forming sterol (ergosterol), and due to incomplete synthesis, cells become permeable. On the other hand, echinocandins inhibit the synthesis of important polysaccharides (1,3-β-glucan) responsible for developing the cell wall, whereas polyenes directly bind to the ergosterol and move inside the cell through the cell membrane by creating pores, and through these pores, cellular organelles come out that cause the death of the cell [16]. While the topical antifungal drugs act on the different sites to target the molecules for the treatment of fungal infections, they show various side effects (Figure 1) on the application site, such as burning, redness, and some allergic reactions [17].

Due to the immediate release of the drug, treatments for an extended period are sometimes needed due to low penetration. Additionally, these drugs may not reach the target location, which could lead to incomplete clearance of the infection. To overcome this problem, the use of natural plant extracts and oils as antifungal agents could be a practical approach [18]. Several plants such as *Bucida buceras* (black olive tree), *Breonadia salicina*, *Harpephyllum caffrum*, *Olinia ventosa*, *Vangueria infausta*, and *Xylotheca kraussiana* have been explored for their antifungal efficacy [19]. On the other hand, due to effective antifungal activity, cinnamon, anise, clove, citronella, peppermint, pepper, camphor essential oils have been used in the formulation of antimycotic drugs [20]. However, the synthesis of an antimycotic drug involves a variety of processes, including high-heat and high-temperature treatments, and as a result of these treatments, the structure of the phytochemicals in the herbal extract is disrupted, leading to the epimerization process. Several studies have found that combining high temperatures and an alkaline state causes structural changes in polyphenolic components. [21,22,23]. As drug carriers, various approaches such as liposomes, ethosomes, trans-ferosomes, niosomes, spanlistics, nanoemulsions, and nanohydrogels are used to overcome this problem. Among all, polysaccharide-based nanohydrogel is an emerging technology, as it shows the same flexibility as natural tissue, with the lowest chance of rejection, minimal side effects, and maximum advantages; for instance, it can target the desired site with controlled release of the antifungal component inside the tissue due to its high penetration power [24]. It can be defined as a three-dimensional nano-sized porous structure with several unique properties, such as high stability, solubility, biodegradability, and biocompatibility with bioactive compounds [5,25]. These gels are macromolecules that can hold a large amount of water and swell up without dissolving due to crosslinking between polymers, which increases their surface area [26]. Therefore, polysaccharide-based nanohydrogels embedded with natural plant extracts and oils have become the primary choice of pharma scientists. These gels protect plant-based bioactive compounds and are effective delivery agents, as they release multiple bioactive compounds in the targeted area. Nanohydrogels can be applied to infected areas, and due to their spreadable nature and penetration power, they get directly absorbed through the skin and reach up to the third layer quickly, thereby effectively reducing the fungal infection. Therefore, in this review, we explain in detail various skin fungal infections, possible treatments, and the effective utilization of plant extract and oil-embedded polysaccharide-based nanohydrogels.

## 2. Skin Fungal Infections and Causative Agents

Fungal exposure causes tissue necrosis of epidermal layers and can lead to superficial, systemic, and subcutaneous mycosis [27]. Except for the production of protease enzymes by yeast and non-dermatophytes, dermatophytes, yeast, and non-dermatophytes share the same pathologic pathway. They always act upon the skin, nails, and hair keratin of human beings and animals, as they utilize keratin as a nutrient source, which ultimately leads to tissue necrosis [28]. According to the natural epidemiological standpoint, dermatophytes can be classified into three major categories: anthropophilic, zoophillic, and geophilic (Table 2).

According to etiology, these can be classified into three genera, viz. *Epidermophyton*, *Microsporum*, and *Trichophyton* [29,31].

Anthropophillicorganisms are a group of dermatophytes predominantly parasitic to humans; they are transmitted from animals and cause dermatophytosis. Zoophilic organisms are parasites to lower animals and are transmitted to humans through direct or indirect contact, whereas geophilic organisms are dermatophytes that are mainly present in the soil as saprophytes; exposure can lead to skin infection or, if severe, can cause systematic infections [27]. Epidermophyton is the species whose microscopic analysis shows between 1 and 9 thin to thick smooth-walled septa; known species are *Epidermophyton floccosum* and *Epidermophyton stockdaleae. Microsporum* can cause skin and hair fungal infection; however, it cannot cause nail fungal infection. Due to the presence of keratin tissue, *Trichophyton* can cause infection in all three areas: nails, hair, and skin. [30]. Moreover, genus *Candida* can cause severe fungal infections such as candidiasis [32]. Twenty species of *Candida* are responsible for human skin infections, and the most common species is *Candida albicans* [33]. These species can cause superficial and systematic types of fungal infections, while dermatophytes can cause superficial types of fungal infections. Primarily, they are found alive on the human skin surface; however, overgrowth of these species can cause severe oral thrush and genital yeast infection [34]. Furthermore, several *Candida* species implicated in human infections are classified as standard, less common, and rare species. *Candida albicans*, *Candida glabrata*, *Candida tropicalis*, *Candida parapsilosis*, *Candida krusei*, *Candida guilliermondii*, *Candida lusitaniae*, and *Candida kefyr* are the examples of common species of *Candida* that are associated with infections in humans. Hyperkeratosis and epidermal hypertrophy persistent with inflammation are diagnostic symptoms for the fungal infection caused by candida species [35].

**Figure 2:**
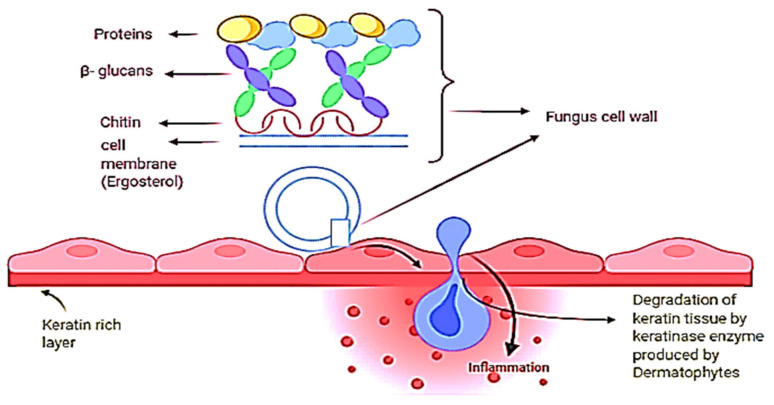
A possible mechanism for dermatophytosis. Dermatophytes are the fungi that need keratin for their growth; therefore, when a fungus cell invades the skin, it produces keratinase, an enzyme that feeds on the skin layer keratin, due to which keratin tissue degradation occurs and causes skin inflammation.

Sporotrichosis is a fungal infection caused by the fungus *Sporothrix*, which is also known as “Rose Garden Disease” [36]. This fungus takes growth in soil, plant matter, and skin. Minor cuts or wounds that come in contact with fungus-enriched soil and plant matter can lead to severe infection. Domestic animal bites such as bites from cats and dogs are also the cause of infection [37]. This type of fungus can cause three types of sporotrichosis, such as lymphocutaneous, fixed, and multifocal or disseminated cutaneous sporotrichosis on the mammal’s skin. Lymphocutaneous sporotrichosis is a common type of cutaneous sporotrichosis in which a small nodule is present at the infectious site; after that, a string of this nodule passes towards the proximal lymphatics with different morphology and surfaces such as with a smooth, watery surface like ulcers [38]. In lymphocutaneous sporotrichosis, fixed cutaneous sporotrichosis is uncommon. In this type of fungal infection, nodule formation at injury sites is common but cannot spread; it remains and develops at the origin. On the other hand, dermatophytes can cause several and severe types of fungal infections because they can cause infection on the innermost layer of human skin (Figure 2).

Tinea capitis, a fungal infection commonly found in preadolescent children, is caused by the dermatophyte species of *Microsporum* and *Trichophyton* [39]. *Epidermophyton floccosum* and *Trichophyton concentricum* are two species that are unable to cause this fungal infection. Tinea capitis born due to direct contact with an infectious person or by sharing utensils with infected people [40]. When sebum production is low, it reduces fatty acids and increases the pH of the scalp, which enhances colonization and results in fungal disease by dermatophytes (tinea capitis koreon) [41]. It is characterized by primary symptoms such as blemishes, blisters, or nodules on the scalp. If not treated well at this stage, it can lead to secondary infections such as superficial reddening of the skin and scaling (dry skin). Fungi enter the hair shaft through the outer root sheath of the hair follicle [42]. The hair shaft comprises three layers, the cuticle (which is the outermost layer of hair that protects the inner structure of hair), the cortex (which is the middle layer that provides color, strength, and texture to the hairs), and the medulla (which is the innermost layer, also known as the marrow of the hair) [43]. When the fungus invades the hair shaft, all these above activities are inhibited and result in Tinea capitis infection; however, sometimes it is non-inflammatory (not permanent hair loss), while sometimes infection is inflammatory (painful nodules with pus and permanent hair loss). It typically affects children of 3 to 7 years of age [44]. Treatment of Tinea capitis uses griseofulvin, terbinafine, itraconazole, and fluconazole. *Penicillium* produces griseofulvin, which can be divided into two micronized and ultra-micronized antifungal drugs for better absorption. The FDA approved intake for the 2-year-old children is 10/mg/kg/day, its micronized type is 20–25 mg/kg/day and ultra-micronized is 10–15 mg/kg/day for 6–12 weeks [45]. It is very effective against *Microsporum* species and shows resistance to *Trichophyton tonsurans*. Griseofulvin reaches the infectious site through sweat glands, and it shows some side effects such as headaches and gastrointestinal upset. Furthermore, terbinafine is an allylamine derivative with fungicidal properties, and it inhibits the enzyme epoxidase, which produces ergosterol. [46]. It is lipophilic; therefore, it can reach the hair follicle, skin, and nails. When taken orally, it binds to the plasma protein and takes 2 h to attain peak plasma value [47]. Itraconazole belongs to the azole class, having 3 nitrogens and one cyclic, heterocyclic ring, which shows fungicidal properties by inhibiting the production of ergosterol; it interferes with the 14-α-demethylase enzyme that converts lanosterol to 14-dimethyl lanosterol [48]. It is a lipophilic drug and can reach the keratinous tissue; however, the time taken to reach the tissue depends upon the dosage, and the optimum time is 4 h. The recommended dose varies in a period for different species; for instance, it is 5 mg/kg/day for 2–4 weeks for *Trichophyton tonsurans* and 4–6 weeks for *Microsporum canis*, and infants use this dose for 3–6 weeks. Stomach pain, diarrhea, and rashes are the side effects of this drug [49]. Fluconazole is an antifungal drug with low molecular weight and high solubility in water that does not require plasma protein to travel to the plasma, which it reaches within 1–2 h after oral consumption. Fluconazole’s efficiency in treating disease is the same as griseofulvin. Gastrointestinal distress and headache are the side effects of it [50].

Tinea corporis is commonly known as ringworm, and the responsible species for this infection are *Trichophyton rubrum*, *Trichophyton tonsurans*, and *Trichophyton mentagrophytes* [51]. In this infection, dermatophytes penetrate the hair follicle and produce scaly ring-shaped itchy rashes on the skin. These ring vesicles are brown in the center and red around the edges, and they mostly appear on the exposed skin. If not treated in its initial stage, kerion-like secondary lesions appear in which infected rings merge and multiply and puss-filled scars develop near the rings. It is spread by conidia, which are spread through hair loss or direct contact with an infected person [52]. Tinea pedis, which is also known as athlete’s foot, occurs by the growth of fungus on the feet in warm and moist conditions. Tinea pedis forms a fluid-rich layer and cause more severe infections if the fluid releases. In moist areas, the fungus spreads by direct contact with infected skin. Tinea pedis is mainly found in people who wear shoes for an extended time and among those who live in industrial areas [53]. *Trichophyton interdigitale*, *Trichophyton tonsurans*, *Trichophyton rubrum*, and *Epidermophyton floccosum* are the main causative agents of tinea pedis which is the fungal infection that occurs in interdigital form and mainly consists of pain-causing scales and ulcers, found between the third and fourth toes [54,55]. Antifungal creams are used for four weeks to treat this fungal infection. However, interdigital tinea pedis requires only one week. Topical antifungals used to treat are azoles, allylamines, butenafine, coclopirox, tolnaflate, and econazole nitrate [56]. At the same time, terbinafine or neftifine(allylamines) show a more effective peak than the azoles. For *Trichophyton* species treatment, a luliconazole formulation is used twice a day for 1–2 weeks [57]. Tinea barbae, also known as barber’s itch and can affect the face’s beard area in two ways: deep and superficial. Zoophile dermatophytes, *Trichophyton mentagrophytes*, and *Trichophyton verrucosum* are the main causative agents of the deep type of this fungal infection which is more severe than the superficial type that is primarily found in farmers. In contrast, crusted patches caused by *Trichophyton rubrum* and *Trichophyton violaceum* species are used to diagnose superficial infection [58]. Tinea faciei is a fungal infection that causes pustules and scales in the non-beard region of the face, causing itching and rashes when exposed to sunlight. Zoophilic dermatophytes such as *Microsporum canis* can cause more severe infection than anthropophilic dermatophytes by causing a reaction under the stratum corneum by producing keratinases, or keratin-degrading enzymes [59]. Tinea manuum is somehow similar to tinea pedis and is diagnosed by dry, scaly patches on the palms and in interdigital areas of the hand. Mainly, *Trichophyton rubrum*, *Trichophyton mentagrophytes*, and *Epidermophytes* can cause this fungal infection [60]. Tinea cruris, also known as jock-itch, is characterized by the patches produced by *Trichopyton rubrum* species that infect hair follicles of the inguinal and perineal area by causing irritation and burning [61]. Treatment of tinea cruris usually lasts 2–4 weeks; however, treatment length can vary depending on the severity of the condition [62]. Tinea versicolor, a superficial infection of the skin, causes hyperpigmentation of the neck, also known as pityriasis versicolor, which is caused by the fungus *Malassezia* species, commonly found on human skin [63]. There are fourteen species found, with *Malassezia furfur* and *Malassezia globasa* being the main causatives [64]. Both topical and oral medications containing specific and nonspecific antifungal agents are used for treatment, with topical treatment serving as first-line therapy and oral treatment serving as second-line therapy for severe infections [65]. Nonspecific agents including sulfur plus salicylic acid, selenium sulfide 2.5%, and zinc pyrithone help remove the dead tissue of fungi from the skin to stop the further invasion of the fungus [66]. In contrast, antifungal agents such as imidazole and allylamine are used to stop the fungus’ mechanism and cure the infection. Ketoconazole is the most effective imidazole topical form of treatment and is available as a cream or a foaming solution [67]. Itraconazole and fluconazole are also used; however, terbinafine is not a viable treatment option for pityriasis versicolor [68].

## 3. Fungal Infections of Nails (Onychomycosis)

Onychomycosis is known as tinea unguium and is a fungal infection of nails in which foot toenails are more affected than fingernails. Onychomycosismainly occurs after tinea pedis, through which the transmission of the fungus takes place [69]. The microbiology of onychomycosis (Table 3) is that dermatophytes, yeast, and non-dermatophytes can cause nail fungal infection; however, dermatophytes participate more and cause nail dysfunction and pain [70].

Patients suffering from peripheral vascular immunologic disorder and diabetes are more prone to onychomycosis. On the other hand, others such as smokers and persons sharing bathing facilities can also face this infection [71].

## 4. Oral and Topical Antifungals and Their Use

According to Table 4, oral or topical antifungal drugs or a combination of both can be used to treat fungal infections caused by dermatophytes [72]. All are aimed at degrading the cell wall of the fungus to inhibit its mechanism of infection and to cause cell death. Topical antifungals are commonly thought of as a first-line treatment for dermatomycosis because as creams, liquids, or sprays they are treatments that can be directly applied to the skin, nails, hair, or even to the mouth. Topical antifungals are more effective than systemic treatment, and their method of administration gives them the advantage of curing skin diseases by their direct application at the site of infection [73]. Candida infections, pityriasis versicolor, tinea barbae, tinea capitis, tinea corporis, tinea cruris, tinea faciei, tinea manuum, tinea nigra, and tinea pedis are examples of fungal infections that are treated with both first- and second-line topical antifungal drugs, depending on the severity of the infection [74,75].

The primary class includes azoles, polyenes, and benzylamine. On the other hand, in addition to drugs in the primary class—such as clotrimazole, econazole, ketoconazole, miconazole, and trioconazole—the drugs terbinafine and amorolfine are also used for the treatment of fungal infection. Ketoconazole shampoo is used to treat fungal infections on the scalp [81]. Antifungal injections are also available, such as amphotericin, flucytosine, itraconazole, voriconazole, anidulafungin, caspofungin, and micafungin, which are also used depending on the type of fungal infection. Antifungals are different from antibiotics because antibiotics can only kill bacteria [82].

Imidazoles and triazoles are the two types of azole antifungals. Both work with the same mechanism, which is by stopping the conversion of lanosterol to ergosterol by inhibiting the work of enzyme lanosterol 14-alpha-demethylase, thus causing porousness in the fungal cell wall [74]. However, both categories show their activity at the different points of the spectrum and have structural differences in the number of nitrogen atoms: in imidazole, it is two, whereas triazole has three nitrogen atoms in it [83]. The polyene antifungals are nystatin, natamycin, and amphotericin B; they are effective against common fungal infections such as candidiasis, aspergillosis, mucormycosis, and cryptococcosis. Polyene antifungals bind to ergosterol, which is the main sterol present in the cell membrane, and they form a polyene–ergosterol complex that creates pores that increase cell permeability [84,85]. On the other hand, amphotericin B shows fungicidal activity for *Candida* species, *Histoplasma capsulatum*, *Cryptococcus neoformans*, *Blastomyces*, and *coccidioides immitis*, although effective treatment depends on parameters such as drug amount and pH (6.0 to 7.5) [86]. Nystatin belongs to the polyene antifungal group and is effective against the mucosal and cutaneous infections caused by candida species, although it is less effective against dermatophytes [87]. Butenafine and allylamines are types of benzylamine drugs and are used topically for the treatment of dermatophytosis. They disturb the cycle of ergosterol synthesis by inhibiting squalene epoxidase enzyme synthesis. Allylamines are considered less effective antifungal agents; however, they have an advantage in the treatment of tinea pedis [88]. When the infection reaches a more severe level, second-line treatment with oral antifungal drugs such as griseofulvin, itraconazole, fluconazole, and terbinafine are among the medications mainly used, with allitraconazole being the most effective [89,90].

## 5. Candidiasis Treatment by Both Oral and Topical Antifungal Treatments

Antifungal medications such as polyenes, azoles, and echinocandins are used to treat candidiasis in both topical and oral forms, depending on the severity of the infection. Nystatin and amphotericin B from polyene; miconazole, clotrimazole, itraconazole, ketoconazole, fluconazole, voriconazole, and econazole from azoles; and caspofungin, micafungin, and anidulafungin from echinocandins are used [91]. These are cyclic hexapeptides with an N-acyl aliphatic or aryl side chain that aid in the treatment of fungal infections caused by Candida and Aspergillus species by disrupting the fungus’ cell wall structure [92]. Echinocandins named caspofungin, micafungin, and anidulafungin are mainly used and these are lipopeptides that act as an inhibitor of the β-d-glucan enzyme (the main component of cell walls) [93]. Therefore, the inhibition leads to cell death and helps to prevent the infection. The pathogenicity of candidiasis depends upon the host strength or factors by which yeast can multiply [94]. Upon candida infection, most microorganisms gather and form a three-dimensional structure on the surface, which is called biofilm [95]. These biofilms are resistant to amphotericin B and fluconazole antifungal drugs. However, the biofilm of *Candida albicans* is more pathogenic than all other species. For a long time, contaminations caused by *Candida* species were treated with azoles, the most common class of antifungal medications. As of late, protection from azoles has expanded in Candida species, both in clinical settings and in vitro. Azoles can treat infection by interfering with the catalyst lanosterol 14-α-demethylase [96]. This catalyst engages with the biosynthesis of a critical component, ergosterol [97]. Azoles mainly target components such as chitin and glucan, which are not present in human skin due to the structural difference between ergosterol and cholesterol [98]. Previously azoles and echinocandins were considered effective drugs against *Candida* species. However, nowadays, these species have become resistant to these drugs [99].

## 6. Resistance to Antifungal Drugs

Fungi have developed a variety of adaptation mechanisms, including cell reactions, drug resistance, and stress reactions. [100]. They can cause a change in osmolarity and drug exposure, interfere with the cell wall, and interrupt the drug-acting pathway. Due to these mechanisms, antifungal drugs show resistance to some fungal species and cannot cure the disease [101]. To withstand cellular pressures such as those generated by drug exposure, fungi must be able to detect and respond to changes in the environment. They have evolved a variety of adaption processes, including complicated circuitries involving cellular responses, such as signaling molecules’ interactions, stress responses, and drug resistance. In this context, Michaelides et al. (1961) considered griseofulvin resistant against dermatophytes. In the 1980s, azoles were reported as antifungal drugs resistant to candidiasis. Mukherjee et al. (2003) *demonstrated the resistance of Trichophyton rubrum* species to terbinafine in onychomycosis-infected patients [102].

### 6.1. Mechanism of Drug Resistance

#### Drug Efflux

Efflux cell membrane transporters are proteins that attach to a variety of substances, including antifungal medicines, and then expel them from the cell. Treatment failure is linked to the development of these molecular pumps, which operate as a protective mechanism against the drug’s cytotoxic effects by limiting the accumulation of hazardous chemicals [103]. By reducing the expected effect in vivo, efflux cell activity causes a gradual and non-specific rise in drug resistance. As a result, increased drug export is one of the most important strategies employed by fungi to develop resistance to antifungal treatments. The pathogenicity of fungal resistance is aided by the mutation of efflux transporters, which favors the emergence of dermatophytosis instances by giving the fungus a colonization advantage [104]. MDR is caused in part by the overexpression of genes from the ATP-binding cassette superfamily (ABC), which has been found in both prokaryotes and eukaryotes throughout evolution. These proteins have two different regions: a nucleotide-binding domain that is extremely conserved and a transmembrane domain that is extremely changeable. ATP-binding cassette transporters actively convey a wide variety of structurally and chemically unrelated substances across membranes by binding and hydrolyzing ATP, decreasing drug accumulation even in cancer cells [105]. MDR, MDR-associated protein (MRP), and the pleiotropic drug resistance (PDR) families are three well-studied families involved in the efflux of hazardous chemicals. The genome sequences of many dermatophytes have revealed a homogeneous group of these transporters with very little genetic variability and only a few distinctive genes in each species studied. Martinez-Rossi et al. (2018) studied about seven species; despite the morphological and adaptive diversity of each species, these dermatophyte genomes discovered a substantial number of encoded ABC transporter domains, with many of the related genes having analogs in all analyzed species, implying that ABC transporter genes work similarly. In the *Trichopyton interdigitale* H6 strain, evidence implicating drug efflux as a mechanism of resistance has been thoroughly examined (previously identified as *Trichophyton rubrum*). Due to reported resistance to both GRS and tioconazole in vitro, the occurrence of a drug efflux phenomenon was initially suggested. Following that, the mdr1 and mdr2 genes were discovered to have elevated transcription levels after exposure to several antifungal drugs. The deletion of the mdr2 gene made a mutant more susceptible to terbinafine (TRB) but not to other medications tested, ruling out the possibility of the mdr2 gene playing a modulatory role in drug sensitivity. When challenged with antifungal medicines, a gene expression study revealed that the mdr4 gene is somewhat dependent on the MDR2 transporter, implying the existence of network connection about MDR activity as well as the reliance of distinct ABC transporters in drug efflux. Different ABC transporters with overlapping substrate profiles may have a wide range of substrate affinity and transport capacity, as well as behave differentially to cellular drug concentrations, affecting the substrates’ actual fate. For example, transcriptional profiling of the pdr1, mdr2, and mdr4 genes in four Trichophyton species—*Trichophyton equinum*, *Trichophyton interdigitale*, *Trichophyton rubrum*, and *Trichophyton tonsurans*—did not reflect the intrinsic phylogenetic relationship among these fungi, nor did it reveal a functional correlation between species and efflux modulation under the tested conditions. The evaluated genes, on the other hand, appear to work in concert, leading to the conclusion that one mdr gene may be compensated by others in terms of extrusion activity [106].

### 6.2. Mutations Affecting Drug Target Genes

In this context, Robbin et al. (2017) demonstrate that mutations in the drug target gene that impairs drug binding and efficacy are one of the most common methods by which microorganisms evolve resistance to drugs. This is a common method by which azole resistance emerges in *Candida albicans*. In clinical isolates of *Candida albicans*, many mutations in the azole target gene ERG11 have been discovered, and they are frequently found in hot-spot regions near the enzyme active site. As *Candida albicans* is a diploid organism, mutations in ERG11 frequently result in the loss of heterozygosity, conferring higher levels of azole resistance via increased dosage of the resistance allele. Mutations in the drug target gene are frequently linked to azole resistance in *Candida neoformans* and *Aspergillus fumigatus*. Resistance to echinocandins is generally caused by mutations in the drug target gene, which are frequently concentrated in two different areas. In *Candida albicans*, changes in FKS1 are frequently followed by a loss of heterozygosity, resulting in two mutant alleles with reduced affinity for the antifungal agent [107]. On the other hand, Khurana et al. (2019) studied Erg11, a gene that codes for 14-lanosterol demethylase. In yeasts, overexpression of gene products and mutations in Erg11 have been documented as mechanisms of azole resistance, but these have yet to be described in dermatophytes. As a result, some mutations affect the hydrogen bond between the medication and the target protein, lowering the affinity of short-tailed triazoles but not long-tailed triazoles, according to a recent study [108].

#### 6.2.1. Decreased Concentration of Drug within Fungi

Increasing the drug efflux mechanism helps to reduce drug buildup within the fungal cell. Membrane proteins called multidrug efflux transporters are found in all living species. These proteins bind to a wide range of structurally and chemically different substances and actively expel them from cells. The medication accumulates less in the cell when the genes encoding these efflux pumps are mutated (upregulated or overexpressed). Antifungal medicines are affected by a variety of efflux mechanisms. The ATP-binding cassette (ABC) superfamily and the main facilitator superfamily are two drug efflux mechanisms that modulate azole resistance. In the *Trichopyton rubrum*, the genes TruMDR1 and TruMDR2 are overexpressed [105,109].

#### 6.2.2. Drug Detoxification

When fungi are exposed to cytotoxic medicines at sub-inhibitory doses, a large number of genes involved in cellular detoxification are activated, contributing to increased drug tolerance. RNA-sequencing (RNA-Seq) examination of the effect of acriflavine (ACR) on *Trichophyton rubrum* revealed that genes involved in cellular detoxification, such as those encoding catalases that protect cells from oxidative stress and reactive oxygen species, were considerably upregulated. Increased production of catalase genes could be a compensatory mechanism to keep the intracellular level of this enzyme stable, sparing cells from the drug’s apoptotic effects. Acriflavine, on the other hand, inhibits catalase activity in vitro. Furthermore, the RNASeq study of the differential gene expression of T. rubrum challenged with UDA indicated that several antioxidant enzyme genes were upregulated. In addition to catalases, UDA stimulates superoxide dismutase, peroxidases, glutathione transferases, and glutathione peroxidases, resulting in enzymatic oxidative detoxification of the body. A previous report showed that Candida resistance to amphotericin B (AMB) could be linked to these findings. Increased catalase activity is also a factor.

Several azole-resistant species have overexpression of target enzymes, which is a compensation mechanism for ergosterol depletion. In *Candida albicans*, overexpression of erg11 is caused by the duplication of the left arm of chromosome 5, which contains the erg11 gene. The development of chromosome 1 disomies allows *Cryptococcus neoformans* to adapt to high FLC concentrations. This resistance is caused by the duplication of two genes on this chromosome: ERG11, which is the target of FLC, and AFR1, which is an azole transporter in *Cryptococcus neoformans*. Extra copies of the *Aspergillus fumigatus* squalene epoxidase gene also give terbinafine resistance. The resistant phenotype was reversed after the strain was cultivated for several generations without terbinafine, resulting in the loss of plasmids encoding the salA gene, confirming that extra copies of this gene caused resistance. Furthermore, the original strain without any plasmid increased the expression of the native salA gene in response to the terbinafine challenge. Unlike bacteria, which can develop antibiotic resistance by the breakdown of these substances, fungi can develop resistance to antifungal drugs by inactivation or destruction [107]. Given that fungi secrete a vast number of enzymes; similar pathways leading to antifungal resistance or tolerance are likely common. Enzymes that fulfill this role in fungi have been discovered, which as a result might be beneficial in the production of enzyme inhibitors and these inhibitors can be used alone or in combination with traditional therapeutic methods.

#### 6.2.3. Changes in Metabolism to Counteract the Drug’s Effect on De Novo Synthesis of Pyrimidines 

The antifungal medicine 5FCcompetes with typical pyrimidine intermediate metabolites for nucleic acid incorporation. *Candida glabrata* has developed 5FC resistance as a result of a de novo increase in pyrimidine synthesis [110].

#### 6.2.4. Variation in Plasma Membrane Composition

A decrease or complete absence of ergosterol in the plasma membrane caused by mutations in non-essential ergosterol genes is a rare mechanism of resistance among polyene drugs, such as the ERG3 mutation in clinical isolates of *Candida albicans* and the ERG6 mutation in *Candida glabrata*.

#### 6.2.5. Biofilms

Biofilms are sessile microbial populations encased in extracellular polymeric compounds that are resistant to antimicrobials and host defenses. Biofilms can be produced by both *Trichophyton rubrum* and *Trichophyton mentagrophytes*.

#### 6.2.6. Modifications in the Biosynthesis of Ergosterol

Azole medicines’ antifungal efficacy is based on the removal of ergosterol from the fungal membrane and the formation of the toxic result 14-methyl-3,6-diol, which causes growth arrest. Inactivation of the ERG3 gene in the late stages of the ergosterol biosynthetic pathway can result in the entire inactivation of C5 sterol desaturase, as well as cross-resistance to a variety of ergosterols.

## 7. Nanohydrogels

Hydrogels are a polymeric network that has a functional group to absorb water, swell up, and not dissolve again into the water due to the presence of binders or crosslinkers. Capillary, osmotic, and hydration forces are responsible for the collaboration between the polymeric chain system and organic liquids, which contribute to the chain network’s balance and expansion [111]. Nanohydrogels are an application that reaches into wide areas, such as drug delivery systems, 3D networking systems, tissue engineering, and biosensors. Historically, hydrogels have been classified into three generations: first-generation, second-generation, and third-generation [112]. Nanohydrogels’ use in drug delivery is very effective due to their extracellular material, which is somehow similar to the extracellular material of human beings. Nanohydrogels can be synthesized in a variety of ways, including one-step and multi-step procedures. They are classified either as copolymers, homopolymers, interpenetrating networks, and semi-interpenetrating networks based on the method of their synthesis. [113]. The copolymer type requires two types of monomers to bind with the crosslinkers. Monomers such as methacrylic acid, PEG-PEGMA, carboxymethyl cellulose, and polyvinylpyrrolidone with crosslinker tetradimethacrylate are used for the synthesis of copolymer hydrogel by following the free radical photopolymerization mechanism that is used for drug delivery and wound dressing material [114,115,116]. In a homopolymer, one type of monomer is used, wherein the choice of crosslinker depends upon the monomer and the nature of the polymerization reaction. Their use also depends upon the nature of the crosslinker, as (Cretu et al. 2004; Das in 2013) showed that the synthesis of hydrogels from monomers such as poly(2-hydroxyethyl methacrylate), 2-hydroxyethyl methacrylate, and polyethylene glycol with crosslinker polyethylene glycol dimethacrylate for drug delivery systems, contact lenses, scaffolds for protein recombination and gels having crosslinker Triethylene glycol dimethacrylate can be used in wound healing and the production of functional tissues [114,117]. Interpenetrating network hydrogels have a pre-prepared hydrogel in which monomers and initiators are dissolved in a solution and are primarily used for drug delivery due to advantages such as pore size and surface properties that can be modified to control the drug release mechanism. On the other hand, semi-interpenetrating network hydrogels have slow drug-releasing properties with advantages similar to interpenetrating network hydrogels, and they also deliver drugs effectively [113].Polymerization is an essential step in the synthesis of hydrogels and can be accomplished in a single step or multiple steps involving different mechanisms, such as in the presence of benzoin isobutyl ether as the UV-sensitive initiator, template copolymerization, or free-radical photopolymerization. Their products are also classified based on the materials used in their manufacture, the types of crosslinkers used, how they appear physically, the net electrical charge, and how they are affected by both physical and chemical changes. [118]. Physical nanohydrogels are referred to as reversible nanohydrogels due to conformational changes, whereas chemically crosslinked hydrogels are referred to as irreversible hydrogels due to configurational changes. [119]. Reversible and irreversible hydrogels can be created using bulk polymerization, which involves dissolving one or more types of monomers and a solvent such as water, a water-ethanol mixture, or benzyl alcohol to form a transparent polymeric network that swells up in the water and acquires flexibility [26]. Crosslinking polymerization is a thermal process that involves the use of ionic and neutral monomers as well as crosslinking agents. A grafting technique is used to strengthen the weak bulk of the polymerized structure. Sometimes unsaturated compounds such as gamma rays and electron beams are used as initiators for nanohydrogel synthesis [120]. In these types of nanohydrogels, first, macro radicals are produced, which further combine with other polymer chains by covalent bonding, and a crosslinked network structure is formed [26]. Stimuli-sensitive hydrogels are hydrogels that are sensitive to certain environmental changes and respond by changing their form or volume when exposed to certain conditions. The physical stimuli to which they respond include light, pressure, temperature, electric field, magnetic field, and ultrasound; chemical stimuli include pH, redox, ionic strength, CO2, glucose; and biological stimuli include enzymes, antigens, glutathione, and DNA. Based on their source at the time of application to the hydrogels in vivo, these stimuli can also be classified as internal or exterior stimuli. Except for the temperature, which can be considered an external or an internal stimulus, chemical, and biological stimuli fall into the first group, whereas physical stimuli fall into the second. These hydrogels have been termed “smart” or “intelligent” in the sense that they perceive a stimulus and respond by changing their physical and/or chemical behavior, allowing the contained medication to be released.

### 7.1. Mechanisms of Different Stimuli-Responsive Hydrogels

(a) Physical stimuli
  1. Temperature
    Temperature changes fluctuate the polymer-polymer and polymer–water interactions that are responsible for swelling and drug release.
  2. Pressure
    Increased pressure causes swelling, and vice versa. This is because the lower critical solution temperature (LCST) of hydrogels rises with pressure. The temperature below which negative thermoresponsive hydrogels swell is known as the LCST.
  3. Light
    The hydrogel is reversibly changed from a flowable to a non-flowable state when exposed to light (UV and visible light).
  4. Electric field
    Swelling–deswelling is caused by changes in the electrical charge distribution within the hydrogel matrix when an electric field is applied, and this is responsible for on-demand drug release.
  5. Magnetic field
    The application of a magnetic field causes pores in the gel to expand, resulting in drug release.
  6. Ultrasound irradiation
    The drug is released when the ionic crosslinks in the hydrogels are briefly broken by ultrasound waves, but the crosslinks are repaired when the ultrasound waves are turned off. This allows for on-demand medicine delivery.
(b) Chemical stimuli
  1. pH
    The charge on the polymer chains changes when the pH changes, causing swelling and drug release.
  2. Ionic strength
    Change in ion concentration also causes swelling and drug release
  3. CO_2_
    A pH-sensitive hydrogel disc comes into touch with a bicarbonate solution in CO2 sensors. When exposed to CO2, the pH of the solution changes, causing the hydrogel to swell or de-swell, causing a change in pressure, which is a measure of CO2 partial pressure.
  4. Glucose
    In reaction to an increase in glucose concentration, hydrogels swell. The combination generated by glucose and phenylboronic acid causes the hydrogels to enlarge, resulting in insulin release.
  5. Redox
    In a reductive environment (high glutathione concentration = 0.5–10 mM), disulfide links in reduction-sensitive hydrogels cleave in the intracellular matrix, releasing bioactive molecules/drugs.
(c) Biological stimuli
  1. Enzymes
    Enzymes are responsible for hydrogel decomposition and, as a result, drug release. This is termed a chemically regulated drug release mechanism.
  2. Antigen
    When hydrogels detect free antigens, they swell and release the molecule.
  3. DNA
    In the presence of ssDNA, single-stranded (ss) DNA grafted hydrogel probes swell.

Hydrogels show unique physical properties, such as high porosity that allows a gel to be loaded or released with highly active components. As a result, the application of hydrogels in drug delivery systems is of great interest [121] (Table 5).

However, sometimes chemically synthesized or drug-loaded nanohydrogels show some side effects such as itching or rashes at the site of application. As a result, antifungal drug-loaded gels based on polysaccharides can be a more highly effective combination of natural and chemical antifungal compounds over chemically synthesized nanohydrogels [125]. Several polysaccharide-based nanohydrogels containing a wide range of active compound formulations have been reported in various studies, and all the developed formulations revealed potential against various types of skin fungal infection-causing strains (Table 6).

Various reports revealed that some strains of *Aspergillus* species are becoming resistant to azoles and nanohydrogels consisting of azole components that showed less activity against the fungal strains. Therefore, to overcome this problem, various plant-based extracts and essential oils have shown promising results against such pathogenic strains of skin fungal infection. However, environmental stress, high temperature, and oxygen are significant factors that lead to detrimental effects on plant-based extracts and essential oils. Therefore, to overcome this problem, scientists have turned to nanohydrogels as their first choice for transporting and protecting bioactive constituents (Table 7).

Plants have been utilized therapeutically for thousands of years everywhere in the world. Medicinal plants incorporate herbs, herbal materials, and items that contain various pieces of plants or other plant materials that have traditionally been used to combat health disorders [137]. Essential oils and herbal extracts have antifungal properties because their phenolic groups act as the primary antimicrobial bioactive compound [138,139,140,141,142]. Phenolic groups are complex, volatile, aromatic compounds with different chemical structures and are stored in various parts of the plant, in particular tissue such as glandular hairs, oil cells, and oil ducts [143]. They are now well known due to their antimicrobial, germ-killing, anti-inflammatory, and antioxidant properties [144]. Many essential oils and their components, such as eucalyptus oil, clove oil, thyme oil, bitter almond oil, cinnamon oil, tea tree oil, and lemongrass oil, are commonly used and have antifungal activity. However, on the other hand, Tabassum and Hamdani (2014) observed that several plants can be used to treat skin diseases, such as aloe vera (*Barbadosaloe*), the gel of which has properties that act against bacteria and fungi (Table 8). In addition, *Bauhinia variegates*, *Beta vulgaris*, *Brassica oleraceae*, *Calendula officinalis*, *Camellia sinesis*, *Cannabis sativus* can aid in the treatment of viral, bacterial, and fungal infections.

Phytochemical compounds are responsible for the antifungal activity of essential oils. Other factors, such as concentration, dosage, length of treatment, and the primary type of fungal species, have an impact on an oil’s antifungal action [149]. The vulnerability of various pathogens to essential oil dosage is determined by the morphological and physiological characteristics of the hyphae. Sometimes fungal species produce enzymes that oxidize and inactivate oils, and when oils come in contact with high temperatures that heat the phytochemicals, epimerization [22] may occur. As a result, to inhibit these types of problems, their use in nanohydrogel formulations allows them to reach an infectious site, such as the third layer of skin, due to their high penetration power, which is why they are gaining more attention in pharmaceutical medical science. Natural polymeric nanohydrogels have numerous advantages due to the popularity of natural materials in the therapeutic area. In addition, natural polymeric nanohydrogels have various applications—such as use as sensors or in drug delivery—because of their ability to concentrate on controlled change during the delivery of bioactive compounds, even when environmental conditions change. Moreover, natural polymeric nanohydrogels gained a preferred position as compared to engineered materials because they show biodegradability, biocompatibility, inexhaustibility, nontoxicity, low-cost, and expanding application [150]. Hosseini and Nabid studied a basil seed mucilage-based hydrogel for drug delivery in 2020. Active compounds used in disease treatment can be incorporated into nanohydrogels using various methods such as (1) physical entrapment; (2) covalent conjugation; and (3) controlled self-assembly. Nowadays, the controlled self-assembly method is widely known for encapsulating active components or drugs inside the gel [151,152].

### 7.2. Physical Entrapment Method for Incorporation

Noncovalent interactions such as ionic, lipophilic, and hydrogen bonding can be used to entrap drugs within nanohydrogels [153]. The self-assembly of cholesterol-containing hyaluronic acid into nanohydrogel for protein delivery is one example. Injection of this gel containing recombinant human growth hormone (rhGH) into rats resulted in a week of sustained release [154]. Hydrogen bonding is demonstrated in the encapsulation of curcumin into chitin nanohydrogels for skin cancer treatment. Chitin is commonly used in nanohydrogel synthesis due to its high biocompatibility, biodegradability, skin non-irritability, ease of availability, and cost-effectiveness. The cationic charge of chitin and the lipophilic nature of both chitin and curcumin help in skin penetration. On the other hand, nanohydrogels are hydrophilic, so the hydrophilic-lipophilic balance of chitin–curcumin nanohydrogels is advantageous. Curcumin interacts through its terminal-OH group with-NHCOCH_3_ of chitin [155].

### 7.3. Covalent Conjugation Method for Incorporation

Nanosystems offer a convenient drug delivery platform. This is because their inherent functional groups play a role in determining the structure and properties of nanoparticles. The drug is covalently conjugated to the crosslinked nanohydrogels, which gives the encapsulated drug additional stability [156]. Polysaccharides contain hydroxyl groups that easily interact with hydroxyl groups formed by forming ester bonds with the drug’s carboxyl groups. Such a drug would be released prematurely in this case due to the cleavage of functional groups by enzymes such as esterase.

### 7.4. Controlled Self-Assembly Method for Incorporation

The term “self-assembly” refers to the autonomous organization of components that results in a good structure. Many molecules self-assemble, which is characterized by diffusion, and the subsequent precise binding of molecules occurs by non-covalent interactions, hydrophobic associations, or by including electrostatic interactions. Due to these a lot of interactions show weaknesses and dominates the assembly’s structure and conformational behavior. In the presence of oppositely charged solutes, polyelectrolyte-based nanohydrogels tend to self-assemble. In an aqueous environment, amphoteric molecules self-assemble into nanoparticles, allowing for improved drug interaction and release from the nanohydrogels. The hydrophilic part of the drug molecule should be exposed to polar or aqueous media, while the hydrophobic part should be fixed in the component’s core. The concentration of polymer above which chains aggregate is referred to as critical micelle concentration or critical aggregate concentration [156,157].

## 8. Antifungal Mechanism of Essential Oil and Plant Extract-Based Nanohydrogel

**Figure 3:**
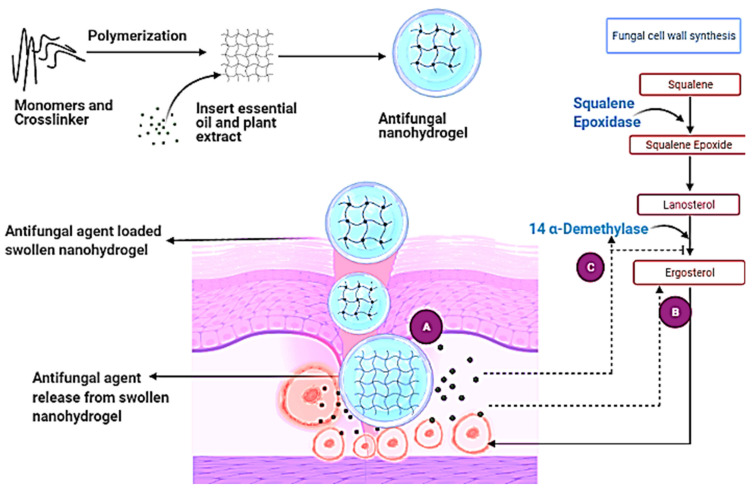
Mechanism action of nanohydrogel and antifungal components. **A:** Swollen nanohydrogel contacts stimuli in the environment and releases antifungal agents by following three mechanisms; **B:** antifungal agent binds to a vital component of the fungus cell wall, create spores, and leads to the death of the cell or causes respiration inhibition of the fungus cell; **C:** antifungal agent interferes with the catalyst and inhibits the ergosterol synthesis from lanosterol, causing cell death.

Essential oils and plant extracts exhibit effective antifungal activity; however, several limitations inhibit the utilization of essential oils and plant extracts [100]. Scientists already revealed that nanohydrogels could pass through the three tissue layers of skin and could thus reach the dermis layer because of their properties (Figure 3). The pH of nanohydrogels is similar to that of human skin; however, when infection occurs, it causes changes at the infectious site. As a result of these changes—and according to the nature, concentration, and hydrophilicity of the polymer used to synthesize the nanohydrogel—a swollen nanohydrogel can receive stimuli to release its antifungal agent through three mechanisms: (1) a diffusion-controlled mechanism; (2) a chemical-controlled mechanism; and (3) a swelling-controlled mechanism [121].

Mainly the nanosized gels release active compounds with the help of diffusion. In a diffusion-controlled mechanism, the gel comes in contact with a high temperature, which leads to an increase in the hydrophobicity of the structure and causes a slow and controlled release of the antifungal agent from the gel, which moves across the concentration gradient, such as from high to low concentration, as calculated by the Fick’s first law of diffusion [158]:

dQ/dt = ADK_d_(Co − C)/h (1)

where dQ/dt is the rate of mass transfer, A is the surface area of film, Kd is the partition coefficient, h is the thickness of the hydrogel, and Co and C both are the concentrations of the drug or active compound at nanohydrogel and infectious site [125]. If the molecular dimensions of the drug molecules are significantly smaller than the pore size of the porous hydrogels, the porosity of the hydrogels is related to the diffusion coefficient of the hydrogels. The release of the drug molecules is hampered by the crosslinked polymer chains when the pore size in the hydrogels and the size of the drug molecules are comparable. The diffusion coefficient decreases as a result. If the rate of drug release exceeds the rate of swelling, the release of the drug is regulated by the swelling-controlled mechanism. In a chemical-controlled mechanism, the active component is released via matrix reactions; this can be an enzymatic, hydrolytic, reversible, or irreversible reaction between the releasing component and the matrix; the releasing action is determined by the rate of crosslinking structure degradation. On the other hand, in the swelling-controlled mechanism, the nanohydrogel starts to swell more when solvent molecules attract towards it, and after this interaction temperature decreases and it leads to the release of the desired component from the polymeric structure. This swelling-controlled mechanism is mainly the function of temperature, in which the transition from swelling to shrinkage occurs [159]. It is also classified as a (1) kinetically controlled release mechanism and (2) reaction diffusion-controlled release mechanism. After the release of the active component, the active component acts against the fungal infection by following different mechanisms, such as an oil that inhibits the growth of a fungus by inserting emulsions in the fungus cell wall where the oil emulsions can cause respiration inhibition and can destroy the cell wall of the fungus hyphae, resulting in cell death, while the oil helps to cure the infection [160]. On the other hand, volatile essential oils inhibit spore formation, such as in a study by Fajinmi et al. (2019) showed the effect of 40 µL *Agathosma betulina* essential oil against the *Trichophyton rubrum* species. These species can produce hyphae that can penetrate the host’s skin and cause an infection inside the skin. The research team subcultured the essential oil-treated fungus on a separate petri plate and observed no fungus growth during and after seven days of an incubation period [143]. Orange oil also acts as an essential oil because of the high content of limonene, myrcene, linalool, and citral components; these show fungicidal effects against a broad spectrum of organisms, such as *Candida albicans*, *Candida krusei*, *Candida tropical*, *Aspergillus niger*, *Aspergillus fumigatus*, and *Penicillium chrysogenum* [161]. Eucalyptus oil acts as both an antibacterial and an antifungal oil [8]. Monoterpenes are the active compounds in eucalyptus oil and work against dermatophytes such as *Microsporum canis* and *Microsporum gypseum* [9].

In this context, Wang and Vonrecum (2011) stated that sometimes nanohydrogels show minimal drug loading efficiency [162]. On the other hand, Vinogradov et al. (2002) revealed that the hydrophilicity of nanohydrogels decreases due to the strong interaction between drug and polymer and causes structural changes, also showing adverse effects due to the presence of surfactant or monomers [67,163]. Hydrogels show significantly less potential for the delivery of hydrophobic drugs. Some of the main disadvantages of the hydrogel are their low mechanical strength, fast dissolution, and low tensile strength [6].

## 9. Conclusions and Future Perspective

Several types of fungal skin infections and their impact on human skin were discussed in detail. The various advantages and applications of antifungal nanocarriers outlined above definitely hold the promise that nanocarriers could potentially revolutionize the pharmaceutical and food sectors. Among all nanocarriers, the scope of nanohydrogels is broad, and formulating functional nanohydrogels for antifungal applications from nature-derived polymers is widely conceived as a sustainable approach that is safer for humans. An array of plant-based biopolymers and essential oils can be incorporated into nanohydrogels for practical components for various skin fungal diseases. A potential mechanism, as well as examples of the successful use of nanohydrogels in the treatment of fungal skin infections, was presented and discussed. Moreover, in the future, concerns over the safety of the polysaccharide-based nanohydrogels will be directly affected by public acceptance of such emerging technologies. In the case of biopolymeric nanohydrogels, the assurance of acceptance comes from the fact that all of the materials discussed here are polymers that, unlike allopathic products, can be quickly degraded in the body. Hence, toxicological studies with nanoformulations are necessary to ensure the effective utilization of nanohydrogels.

## Figures and Tables

**Table 1:** The occurrence of Tinea infection in various body parts.

Tinea Infection	Affected Locations	References
Tinea capitis	Scalp	[7]
Tinea corporis	Trunk	[8]
Tinea faciei	Face	[9]
Tinea manuum	Hands	[10]
Tinea pedis	Feet	[11]
Tinea unguium	Nails	[12]

**Table 2:** Most relevant species of fungal infection according to their natural habitat.

Dermatophytes Based on Their Habitat	Fungal Species Belonging to Different Dermatophyte Group	Infection Site	References
Anthropophillic	*Microsporum audouinii*	Scalp	[29]
	*Trichophyton concenricum*	Body	
	*Microsporum ferrugineum*	Scalp	
	*Trichophyton interdigitale*	Foot, groin, nails	
	*Trichophyton megninii*	Scalp, beard	
	*Trichophyton rubrum*	Foot, nails, body	
	*Trichophyton schoenleinii*	Scalp	
	*Trichophyton soudanense*	Scalp	
	*Trichophyton tonsurans*	Scalp, body	
	*Trichophyton violaceum*	Scalp, body, nails	
Zoophillic	*Microsporum canis*	Scalp, body	[29,30]
	*Microsporum distortum*	Scalp	
	*Trichophyton equinum*	Scalp	
	*Microsporum nanum*	Scalp, body	
	*Trichophyton verrucosum*	Exposed areas	
Geophilic	*Microsporum fulvum*	Scalp, body	[29]
	*Microsporum gypseum*	Scalp, body	

**Table 3:** Most common fungal agents for the treatment of onychomycosis.

Onychomycosis Microbiology	Name of Species Cause Onychomycosis
Dermatophytes	*Epidermophyton floccosum*
	*Microsporum species*
	*Trichophyton interdigital*
	*Trichophyton mentagrophytes*
	*Trichophyton rubrum*
	*Trichophyton tonsurans*
Nondermatophyte	*Acremonium species*
	*Alternaria species*
	*Aspergillus species*
	*Cladosporium carrionii*
	*Fusarium species*
	*Geotrichum cadidum*
	*Lasiodiplodia theobromae*
	*Onychocola species*
	*Scopulariopsiss pecies*
	*Scytalidium species*
Yeast	*Candida albicans*
	*Candida parapsilosis*

**Table 4:** Classification of antifungal components and their effectiveness against various fungal infections.

Class of Antifungal Agents	Antifungal Agents	Chemical Structure of Different Antifungal Agents	Fungal Infections	Desired Treatment Duration	References
Imidazoles	Clotrimazole (1%)	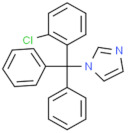	Tinea corporis Tinea cruris Tinea pedis	4–6 weeks	[10]
	Econazole (1%)	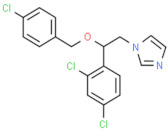	Tinea corporis Tinea cruris Tinea pedis	4–6 weeks	[11]
	Miconazole (1%)	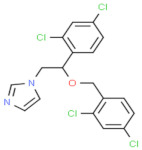	Tinea corporis Tinea cruris Tinea pedis	4–6 weeks	[73]
	Oxiconazole (2%)	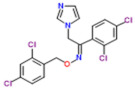	Tinea corporis Tinea cruris Tinea pedis	4 weeks	[57]
	Sertaconazole (2%)	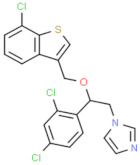	Tinea corporis Tinea cruris Tinea pedis	4 weeks	[11]
	Luliconazole (1%)	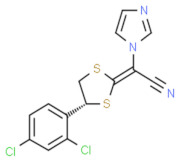	Tinea corporis Tinea cruris Tinea pedis	2 weeks	[54]
	Eberconazole (1%)	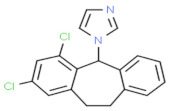	Tinea corporis Tinea cruris Tinea pedis	2–4 weeks	[10]
Triazoles	Efinaconazole (10%)	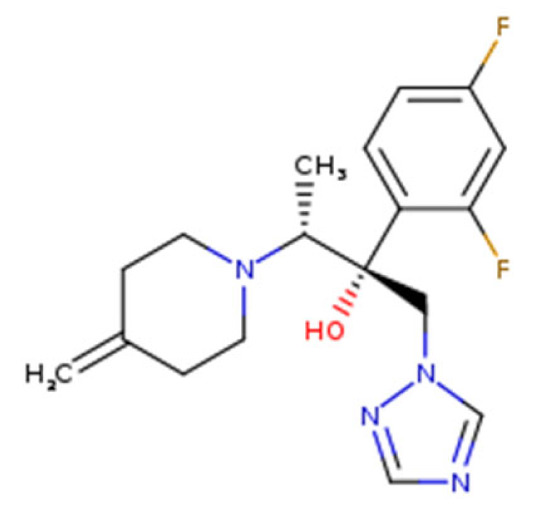	Tinea pedis	52 weeks	[12]
Allylamines	Terbinafine	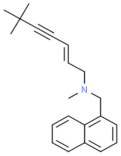	Tinea corporis Tinea cruris Tinea manuum Tinea pedis	2 weeks 2 weeks 4 weeks 4 weeks	[76]
	Naftifine (1%)	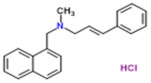	Tinea corporis Tinea cruris Tinea pedis	Used 2 weeks beyond the resolution of symptoms	[77]
	Butenafine (1%)	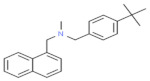	Tinea corporis Tinea cruris Tinea pedis	2–4 weeks	[78]
Others	Amorolfine (0.25%)	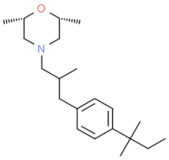	Tinea corporis	4 weeks	[79]
	Amphotericin B (0.1%)	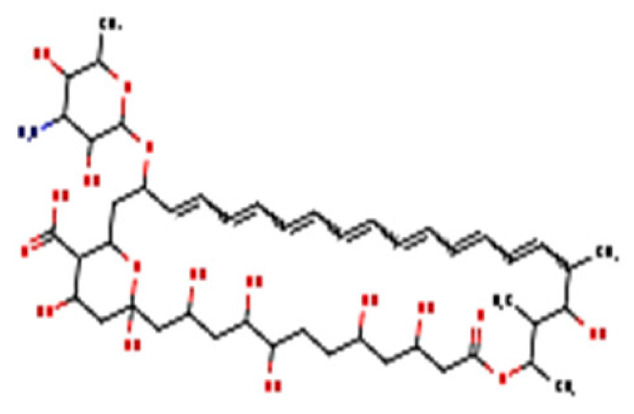	Tinea corporis	4 weeks	[80]

**Table 5:** Clinical trials of hydrogels in the treatment of skin disorders.

Type of Hydrogel	Clinical Study	Agent	Skin Disorder	References
Clindamycin/Tretinoin Hydrogel	Clindamycin/Tretinoin Hydrogel	Combination Of Clindamycin (1%) and Tretinoin (0.025%)	Acne vulgaris	[79]
Liposomal Methylene Blue Hydrogel	Randomized and comparative study of 35 patients (21 men and 14 women) with varying degrees of acne vulgaris on the back	Methylene Blue	Acne vulgaris (Truncal)	[80]
Carboxymethylcellulosebased Hydrogel	Single-blind study on 20 patients (12 men and 8 women)	Resveratrol	Acne vulgaris (Facial)	[122]
Hydrogel Patch	Men and women with plaque-type psoriasislesions	Mometasone Furoate	Psoriasis	[123]
Hydrogel Micropatch	100 psoriatic patients (75 men and 25 women) and 100 healthy volunteers	Mometasone Furoate	Psoriasis	[124]

**Table 6:** Existing antifungal drug-loaded polysaccharide-based formulations.

Polysaccharide	Active Compounds	References
Galan gum/cyclodextrin	Fluconazole	[126]
Galan gum	Terbinafine HCL	[127]
Chitosan/carbopol/natrosol	Terbinafine HCL	[128]
Galan gum/carrageenan	Econazole	[129]
Galan gum/carbopol934P hydroxyl propyl methyl cellulose E50LV	Clotrimazole	[130]
Galan gum/glycerol	Fluconazole	[131]
Galan gum	Natamycin	[132]

**Table 7:** Natural extract entrenched antifungal formulations.

Formulation	Natural Extract	Active Ingredients of the Natural Extract	Effective against	References
Methylcellulose hydrogel	*Melissa officinalis*	citronellal (50%), citronellol (10%), and geraniol (14%)	Candidiasis	[133]
Polyherbal gel	*Piper betal* and *Piper nigrum* leaf extract	methanolic hydro extracts	*Candida albicans*	[134]
Copper chitosan nanocomposite hydrogel	*Thymus vulgaris*	p-cymene, thymol, and 1,8-cineole	*Aspergillus flavus*	[134]
Hydroxypropylmethylcellulose hydrogel	*Melaleuca alternifolia* (Tea tree oil)	terpinene-4-ol	Oral candidiasis	[135]
Carbopol hydrogel	*Cymbopogon citratus* (Lemongrass oil)	geraniol, geranylacetate, and monoterpene olefins	*Candida albicans*	[136]

**Table 8:** Plant extracts having antifungal properties.

Antifungal Plant Extracts	Effective against	References
Leaves of *Piperregnellii*	*Trichophyton rubrum, Trichophyton mentagrophytes, Microsporum canis*	[145]
Roots of *Rubiatinctorum*	*Asperegilus niger, Alternaria lternaria, Penicillium verrucosum, Mucor mucedo*	[146]
*Tithoniadiversifolia*	*Microbotryum violaceum, Chlorella fusca*	[147]
*Daturametel*	*Candida albicans,* *Candida tropicalis*	[148]
*Alliumcepa* and *Alliumsativum*	*Malassezia furfur*, *Candida albicans*, and other *Candida* species	[18]

## Data Availability

Not applicable.
